# A Rapid MALDI-TOF MS Identification Database at Genospecies Level for Clinical and Environmental *Aeromonas* Strains

**DOI:** 10.1371/journal.pone.0048441

**Published:** 2012-10-31

**Authors:** Cinzia Benagli, Antonella Demarta, AnnaPaola Caminada, Dominik Ziegler, Orlando Petrini, Mauro Tonolla

**Affiliations:** 1 Institute of Microbiology, Bellinzona, Switzerland; 2 Mabritec AG, Riehen, Switzerland; 3 Microbial Ecology, Microbiology Unit, Plant Biology Department University of Geneva, Genève, Switzerland; University of Helsinki, Finland

## Abstract

*The genus Aeromonas* has undergone a number of taxonomic and nomenclature revisions over the past 20 years, and new (sub)species and biogroups are continuously described. Standard identification methods such as biochemical characterization have deficiencies and do not allow clarification of the taxonomic position. This report describes the development of a matrix-assisted laser desorption/ionisation–time of flight mass spectrometry (MALDI-TOF MS) identification database for a rapid identification of clinical and environmental *Aeromonas* isolates.

## Introduction

Bacteria belonging to the genus *Aeromonas* are widely distributed in freshwater and brackish environments, and have long been recognized as etiologic agents for fish diseases [1]. They are included into the class Gammaproteobacteria, comprising Gram-negative, non-spore-forming rod-shaped bacteria, are facultative anaerobic oxidase- and catalase-positive, glucose-fermenting, resistant to the vibriostatic agent O/129, and generally motile [2].

*Aeromonas* play also a significant role as opportunistic pathogens for humans causing gastroenteritis, septicemia, pneumonia, meningitis, and wound infections in immunocompetent as well as in compromised patients. *A. hydrophila*, *A. caviae* and *A. veronii* (biovar *sobria* and biovar *veronii*), are clinically the most significant species [3].

So far, the genus *Aeromonas* comprises 21 validly proposed species: *A. allosaccharophila, A. aquariorum, A. bestiarum, A. bivalvium, A. caviae* (synonym: *A. punctata*) *A. culicicola, A. encheleia* (corresponds to HG 11), *A. eucrenophila, A. hydrophila, A. jandaei, A. media, A. molluscorum, A. popoffii, A. salmonicida, A. schubertii, A. sharmana, A. simiae, A. sobria, A. tecta, A. trota* (synonym: *A. enteropelogenes*), *A. veronii* (synonym: *A. ichthiosmia*). It has to be noted that within these proposed species the position of *A. allosaccharophila, A. culicicola and A. sharmana* has to be clarified since the first two might belong to *A. veronii* and the last one seems not belong to the genus *Aeromonas* at all [4], [5].

Several phylogenetic studies on *Aeromonas* allowed the elevation of the genus name to the rank of family [2], [6], [7], [8]. Nevertheless the taxonomy of this genus is rather complex and has been submitted to ongoing changes due to newly described species [9], [10], [11], [12], [13], [14] and rearrangements of existing taxa [15], [16], [17], [18], [19], [20]. One major problem in *Aeromonas* identification relies on the fact that some species are phenotypically very similar (e.g. *A. caviae* and *A. media*, *A. veronii* and *A. sobria*). Several molecular methods have been therefore applied as an alternative to the laborious DNA-DNA hybridization technique for resolving the *Aeromonas* taxonomy and even though the sequence analysis of ribosomal RNA genes allowed for the discrimination of the genospecies [6], [21], [22], other more discriminating housekeeping genes such as *gyrB* and *rpoD* are now increasingly used [8], [23], [24], [25], [26]. Nevertheless, sequencing and phylogenetic methods are costly, time consuming and therefore not appropriate for a rapid species identification in the diagnostic laboratory. A valid alternative to conventional methods of bacterial identification and classification, based on the characterization of biomarker molecules, but definitely more rapid and reliable is the mass spectrometry technique [27]; MALDI-TOF MS (matrix assisted laser desorption ionization mass spectrometry – time of flight) combined with a reliable database is a powerful method for the identification and comparison of microbial isolates based on protein fingerprints analysis of whole cells [28]. MALDI-TOF MS applications in microbiology are important for proteomic and natural product analyses [29]. This technique can be used to detect non-volatile and thermally unstable molecules from a few to several hundred kDa, the most applicable range used for the analysis is 2–20 kDa. The identification of microorganisms by MALDI-TOF MS is based on the detection of mass signals from biomarkers that are specific at genus, species or sub-group level.

All mass spectra were generated in positive linear mode by scanning the sample spot with the laser beam, and after signal acquisition, the raw mass spectra are processed automatically by smoothing, baseline correction and peak recognition [30]. The essential information used for microbial identification is contained in a peak list containing m/z values and intensities. This list is analysed by comparison to the database SARAMIS™ (Spectral Archive And Microbial Identification System), in which the identification at the species level is based on a percentage of confidence referred to reference spectra (SuperSpectra™) that contain family, genus and species specific m/z biomarkers, as described in the SARAMIS™ user manual. For the generation of one SuperSpectra™ some representatives isolates of one species from different locations (hospitals, reference centers and strain culture collections) are needed. Beside the FingerprintSpectra every isolate will be determined by accredited and published microorganism identification procedures. The SuperSpectra™ are generated based on measurements of well known microorganisms and contain sets of genus, species and strain biomarkers which are characteristic for the respective group of microorganisms. Superspecta™ are computed from typical strains covering more than 90% of the intraspecific diversity in most species.

Accuracy of the identification strongly relies upon the robustness of the database and the choice of reference isolates. This is especially important when considering genera comprising species of clinical and environmental origin presenting a high genetic diversity.

There are excellent precedents for the application of MALDI-TOF MS for taxonomic studies [31], [32], [33], [34], as well as for routine diagnostic [35].

Previous studies proved the applicability of this technique for the identification of the *Aeromonas* species [36], [37], [38]. The major aim of this study was to establish a rapid and reliable species identification tool for the genus *Aeromonas* using the SARAMIS™ identification system based on a relatively high number of phylogenetically well characterized isolates of clinical and environmental origin.

## Methods

### Bacterial Strains

92 morphologically and genetically well characterized strains (see supporting information Table S1) belonging to all known genospecies of the genus *Aeromonas* were used to create the m/z reference library system using the SARAMIS™ software. All strains were phylogenetically typed and assigned to the respective genetic species using the housekeeping gene *gyrB.* The obtained sequences were deposited in GenBank and accession numbers are listed in Table S1. The mass fingerprinting identification database produced was then evaluated on 741 clinical and environmental isolates. All strains were grown on Blood Agar at 30°C for 24 hours previous to the protein fingerprinting mass spectrometry analysis.

**Figure 1:**
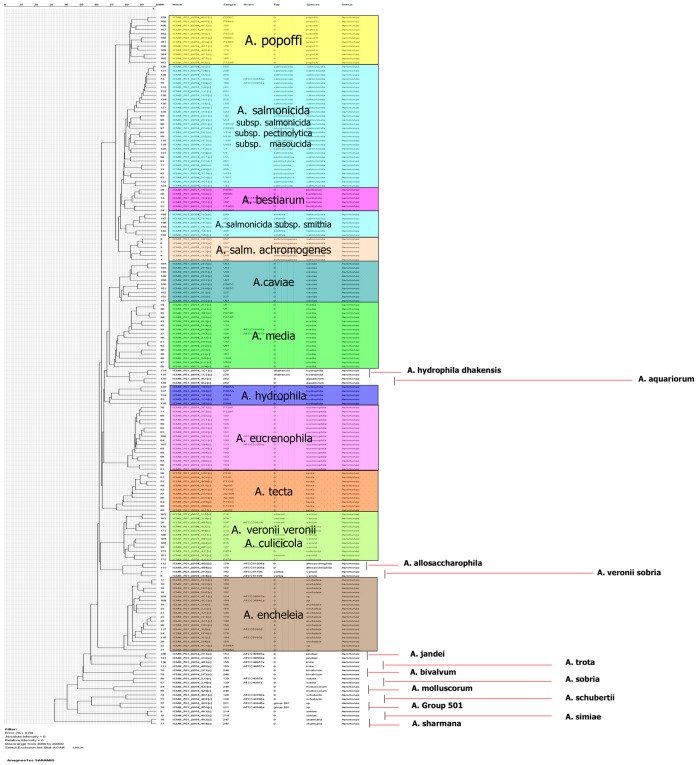
Dendrogram resulting from single-linkage cluster analysis of MALDI-TOF mass spectra. Error 0.08%; Mass range from *m/z* 2,000 to 20,000.

### DNA Extraction

Genomic DNA was extracted from colonies grown on blood agar according to Demarta et al. [39], and resuspended in TrisEDTA buffer (10 mM Tris/HCl, 1 mM EDTA, pH 8.0).

### PCR Amplification and Sequencing

The sets of primers used for amplification and sequencing of the *gyrB* gene have been reported elsewhere [40], [41].

**Table 1:** Characteristic masses retained for the creation of SuperSpectra™.

A. hydrophila	A. caviae	A. popoffi	A. tecta	A. eucrenophila	A. media	A. media	A. media	A. bestiarum	A. encheleia	A. salmonicida	A. veronii	A. veronii	A. veronii	A. sobria
3332	3150	3683	3032	3150	3047	2007	2006	3150	3151	3828	3772	3153	2241	3590
3871	3435	3827	3150	4258	3435	3156	2039	3844	3899	4346	3930	3606	3047	4172
4169	4302	4257	3970	4458	3665	3671	2071	3863	4259	4591	4189	4174	4170	4260
4256	4394	4322	4257	4514	4258	4265	2087	4257	4487	4699	4309	4262	4257	4348
4318	4974	4393	4458	4700	4317	4325	2093	4440	4600	5050	4393	4366	4309	4650
4445	5051	4879	4766	5051	4460	4468	2514	4591	4701	5584	5071	4504	4361	5052
4698	5187	5203	5075	5612	4591	4599	2614	4655	5007	5675	5186	4646	4490	6104
5003	5394	5665	5477	5743	4700	4707	6107	5070	5144	5700	5393	4704	4518	6307
5049	5687	6064	6876	6070	5462	5694	6313	5155	5351	5877	5590	4989	4670	6934
5706	5885	6329	7704	6306	6083	5903	8615	5603	6071	6085	6197	5161	5155	7184
6022	6213	6914	7943	6481	6305	6109	8923	5637	6307	6305	6859	6313	7234	7336
6304	7210	7194	8604	6833	6481	6315	9193	6305	6482	6480	7236	6867	7410	7920
7208	7410	7220	8963	7335	6861	6490	9220	6480	6951	6919	7408	9195	7749	8831
7347	7463	7492	9014	7888	7333	7206	9408	7566	7197	7195	7934	9384	8624	8941
7477	8606	7904	9535	8344	7369	7343	10318	7730	7335	7332	8160	9980	9042	9204
7746	8979	8060	10628	8706	7473	7684	10931	8343	8263	7658	8621	11164	10280	9231
8637	8998	9184	10953	9029	7915	8617	11376	9400	8607	8343	9040	11191		10904
8913	9401	9399	11192	9186	8343	9197	12205	10136	9201	9400	11385	12216		11235
9183	9949	9682	11399	9401	9185	9221		11273	9403	11166		12406		11422
9398	11373	11329	11753	10311	11193	9412			10313	11348				12412
10008			12273	12288	11368	10323			10648					12461
					12197	10937								
						11383								

### Phylogenetic Analyses

Nucleotide sequences of *gyrB* gene (fragment of 1100 bp) was aligned and phylogenetically analysed using MEGA version 3.1 [42].

Phylogenetic tree was constructed using the Neighbour-Joining method with genetic distances computed by employing Kimura’s 2-parameter method [41].

**Table 2:** Identification values at species level obtained with the created SuperSpectra™.

		>99%	90–99%	<90%	NI	n
*Aeromonas*	*hydrophila*	158	5	2	2	**167**
*Aeromonas*	*caviae*	176	6	7	8	**197**
*Aeromonas*	*media*	76	9	3	3	**91**
*Aeromonas*	*tecta*	12				**12**
*Aeromonas*	*popofii*	13			6	**19**
*Aeromonas*	*eucrenophila*	21	3		1	**25**
*Aeromonas*	*encheleia*	8	1			**9**
*Aeromonas*	*bestiarum*	25	5			**30**
*Aeromonas*	*salmonicida*	41	1	1	1	**44**
*Aeromonas*	*veronii*	90	5		8	**103**
*Aeromonas*	*sobria*	21				**21**
*Aeromonas*	*spp*				23	**23**
**n**		**641**	**35**	**13**	**52**	**741**

### MALDI-TOF MS

Strains were transferred from the colony directly on a 48-position stainless steel FlexiMass™ target plate (Shimadzu Biotech, Kyoto, Japan) using a plastic loop. The transferred colony material was then overlaid with 0.5 µl of Matrix (DHB 75%) solution containing 75 mg/ml 2, 5-dihydroxybenzoic acid in acetonitrile/ethanol/water (1∶1:1) supplemented with 3% trifluoroacetic acid. All mass spectra were acquired using an AXIMA Confidence™ (Shimadzu Biotech, Kyoto, Japan) mass spectrometer, equipped with a nitrogen laser (pulse width: 3 ns) operated in positive linear mode. The measured mass range of spectra was 2000–20,000 Da. A minimum of 20 laser shots per sample was used to generate each ion spectrum. For each bacterial sample, 50 protein mass fingerprints were averaged and processed.

All spectra were processed by the MALDI-TOF MS Launchpad 2.8 software (Shimadzu Biotech, Kyoto, Japan).

### Data Analysis

A database identification system was established analyzing 92 morphologically and genetically well characterized *Aeromonas* strains belonging to all known species of the genus. The resulting peak lists of these samples were exported to the SARAMIS™ software package (bioMérieux, France) and submitted to single-linkage cluster analysis to produce taxonomic trees. These trees were compared to a *gyrB* phylogenetic tree (Neighbour-Joining). Specific biomarkers containing sets of genus, species and strain characteristic masses were used for the creation of species-specific SuperSpectra™ recognizing the most frequently encountered species. 11 different SuperSpectra™ were created that allow identifications of: *A. hydrophila, A. caviae, A. media, A. tecta, A. popoffi, A. eucrenophila, A. encheleia, A. bestiarum, A. salmonicida, A. sobria and A. veronii*).

## Results and Discussion

The protein mass fingerprint analysis emerging from the MALDI-TOF MS data of 92 genetically well characterized *Aeromonas* strains provided a good separation at genospecies (Fig. 1) level comparable with the phylogenetic tree obtained by *gyrB* gene sequencing.

In fact both trees clustered the species *A. veronii* (*A. veronii* biovar *sobria*, *A. veronii* biovar *sobria*), *A. culicicola,* and *A. allosaccarophila* together, confirming the hypothesis that this group in fact represents only one genospecies [18].

Interesting the m/z profiles analysis allowed to separate the two biovars *veronii* and *sobria*, furthermore the profile of the strain ATCC 51106 *A. veronii* biovar *sobria* was more closely related to that of *A. allosaccarophila* ATCC 51208 than to that of *A. veronii* biovar *veronii*, confirming the results obtained with the *gyrB* sequences.

Moreover MALDI-TOF MS analysis categorized in a single cluster *A. encheleia* and the unnamed *Aeromonas sp*. HG11 [23] and allowed the segregation in the different genospecies of the *A. salmonicida/A. bestiarum/A. popoffii* group.

*A. salmonicida* and *A. bestiarum* are difficult to separate on the basis of 16S rRNA (differ in only 2 nucleotide positions) [2] but they could be separated using *gyrB* as well as other housekeeping genes such as *rpoB* or *rpoD*.

At the subspecies level, *A. salmonicida* formed a very uniform group, with respective intraspecies substitution rates of 1.3 and 0.8% for *gyrB* and *rpoB*, rendering very difficult to classify strains at the subspecies level [41]. MALDI-TOF MS seemed to allow a better differentiation of the strains in study. The type strains of each subspecies were well differentiated and formed a defined group in the MALDI-TOF MS dendrogram (Fig. 1).

A branch in the MALDI-TOF MS dendrogram groups in one single cluster strains assigned to the species *A. aquariorum* and *A. hydrophila* subsp. *dhakensis* (Fig.1). Data based on phylogenetic analysis by sequencing *gyrB*, *rpoD* and 16S rRNA [43], strongly suggested that strains of *A. hydrophila* subsp. *dhakensis* belongs in fact to the species *A. aquariorum*, confirming the results obtained with MALDI-TOF MS (Fig. 1).

Due to the reliable identification at species level, it was possible to create 11 different SuperSpectra™ for *A. hydrophila*, *A. caviae*, *A. veronii, A. media*, *A. tecta*, *A. popoffii, A. eucrenophila, A. encheleia, A. bestiarum, A. sobria* and *A. salmonicida* to be used for the identification of the strains at the species level (Table 1).

We tested the new SuperSpectra™ with 741 strains of *Aeromonas*. 93% of these strains were successfully identified (Table 2), 93% of them with an identification value greater than 99%.

52 of 741 strains (7%) could not be identified mostly due to the absence of SuperSpectra™ (23 strains, *A. allosaccharophila, A. aquariorum, A. bivalvium, A. culicicola, A. jandaei, A. molluscorum, A. schubertii, A. sharmana, A. simiae, A. trota*), or for the absence of SuperSpectra™ with sufficient coverage in our database (29 strains, Table 2).

These results demonstrate that the mass spectral data of the strains contained sufficient protein information to distinguish between genera, species, and strains (Table 2).

Another mass spectrometry study of intact-cell with *Aeromonas* strains [37] also confirmed that the signals generated from the analysis of the protein masses could be used as specific biomarkers for the differentiation below the species level. For the the majority of the species analysed the identification was successful.

With *A. tecta* and *A. sobria* we obtained a correct identification for all the strains, whereas for *A. eucrenophila*, *A. salmonicida*, and *A. hydrophila* only 1 strains for the first two and 2 strains for the last species could not be identified.

Identification of *A. popoffii* with the created SuperSpectra™ was possible only in 46% of the cases. These failure could be due to insufficient coverage of the specific SuperSpectra™ or lack of performance of the last.

The approach presented in this paper uses the technique MALDI-TOF MS to develop a rapid, sensitive and specific method to detect isolates of the genus *Aeromonas*.

Our work highlighted the importance of testing well characterized strains of different origins for producing high quality MALDI-TOF MS databases as rapid identification tools. In conclusion, we can affirm that MALDI-TOF MS is a rapid and relatively inexpensive method for the identification of *Aeromonas* species and constitutes a valid alternative to conventional methods of identification and classification.

## Supporting Information

Table S1 **Strains used in this study.**
(DOC)

## References

[1] Austin BaA, C (1996) Fish pathogens. The Genus *Aeromonas*. B. Austin, M. Altwegg, P. J. Gosling & S. Joseph ed: Chichester: Wiley. 197–244.

[2] Martin-Carnahan A, Joseph SW (2005) Genus I. *Aeromonas* Stanier 1943, 213AL. In: D. J. Brenner NRK, J. T Staley, and, GMG (eds). Bergey’s manual of systematic bacteriology. 2nd ed. New York, NY.: Springer,. 557–578.

[3] Janda JM, Abbott SL (1998) Evolving concepts regarding the genus *Aeromonas*: an expanding Panorama of species, disease presentations, and unanswered questions. Clin Infect Dis 27: 332–344. 9709884 10.1086/514652

[4] Collins MD, Martinez-Murcia AJ, Cai J (1993) *Aeromonas enteropelogenes* and *Aeromonas ichthiosmia* are identical to *Aeromonas trota* and *Aeromonas veronii*, respectively, as revealed by small-subunit rRNA sequence analysis. Int J Syst Bacteriol 43: 855–856. 8240968 10.1099/00207713-43-4-855

[5] Janda JM, Abbott SL (2010) The genus *Aeromonas*: taxonomy, pathogenicity, and infection. Clin Microbiol Rev 23: 35–73. 20065325 10.1128/CMR.00039-09 PMC2806660

[6] Martinez-Murcia AJ, Benlloch S, Collins MD (1992) Phylogenetic interrelationships of members of the genera *Aeromonas* and *Plesiomonas* as determined by 16S ribosomal DNA sequencing: lack of congruence with results of DNA-DNA hybridizations. Int J Syst Bacteriol 42: 412–421. 1380289 10.1099/00207713-42-3-412

[7] Ruimy R, Breittmayer V, Elbaze P, Lafay B, Boussemart O, et al (1994) Phylogenetic analysis and assessment of the genera *Vibrio*, *Photobacterium*, *Aeromonas*, and *Plesiomonas* deduced from small-subunit rRNA sequences. Int J Syst Bacteriol 44: 416–426. 7520733 10.1099/00207713-44-3-416

[8] Yanez MA, Catalan V, Apraiz D, Figueras MJ, Martinez-Murcia AJ (2003) Phylogenetic analysis of members of the genus *Aeromonas* based on *gyrB* gene sequences. Int J Syst Evol Microbiol 53: 875–883. 12807216 10.1099/ijs.0.02443-0

[9] Pidiyar V, Kaznowski A, Narayan NB, Patole M, Shouche YS (2002) *Aeromonas culicicola* sp. nov., from the midgut of *Culex quinquefasciatus* . Int J Syst Evol Microbiol 52: 1723–1728. 12361279 10.1099/00207713-52-5-1723

[10] Harf-Monteil C, Fleche AL, Riegel P, Prevost G, Bermond D, et al (2004) *Aeromonas simiae* sp. nov., isolated from monkey faeces. Int J Syst Evol Microbiol 54: 481–485. 15023964 10.1099/ijs.0.02786-0

[11] Minana-Galbis D, Farfan M, Fuste MC, Loren JG (2004) *Aeromonas molluscorum* sp. nov., isolated from bivalve molluscs. Int J Syst Evol Microbiol 54: 2073–2078. 15545437 10.1099/ijs.0.63202-0

[12] Minana-Galbis D, Farfan M, Fuste MC, Loren JG (2004) Genetic diversity and population structure of *Aeromonas hydrophila*, *Aer*. *bestiarum*, *Aer*. *salmonicida* and *Aer*. *popoffii* by multilocus enzyme electrophoresis (MLEE). Environ Microbiol 6: 198–208. 14871204 10.1046/j.1462-2920.2004.00554.x

[13] Minana-Galbis D, Farfan M, Fuste MC, Loren JG (2007) *Aeromonas bivalvium* sp. nov., isolated from bivalve molluscs. Int J Syst Evol Microbiol 57: 582–587. 17329789 10.1099/ijs.0.64497-0

[14] Minana-Galbis D, Farfan M, Gaspar Loren J, Carmen Fuste M (2010) Proposal to assign *Aeromonas diversa* sp. nov. as a novel species designation for *Aeromonas* group 501. Syst Appl Microbiol 33: 15–19. 20005654 10.1016/j.syapm.2009.11.002

[15] Pavan ME, Abbott SL, Zorzopulos J, Janda JM (2000) *Aeromonas salmonicida* subsp. *pectinolytica* subsp. nov., a new pectinase-positive subspecies isolated from a heavily polluted river. Int J Syst Evol Microbiol 50 Pt 3: 1119–1124. 10.1099/00207713-50-3-1119 10843053

[16] Huys G, Pearson M, Kampfer P, Denys R, Cnockaert M, et al (2003) *Aeromonas hydrophila* subsp. *ranae* subsp. nov., isolated from septicaemic farmed frogs in Thailand. Int J Syst Evol Microbiol 53: 885–891. 12807217 10.1099/ijs.0.02357-0

[17] Huys G, Kampfer P, Albert MJ, Kuhn I, Denys R, et al (2002) *Aeromonas hydrophila* subsp. *dhakensis* subsp. nov., isolated from children with diarrhoea in Bangladesh, and extended description of *Aeromonas hydrophila* subsp. *hydrophila* (Chester 1901) Stanier 1943 (approved lists 1980). Int J Syst Evol Microbiol 52: 705–712. 12054229 10.1099/00207713-52-3-705

[18] Huys G, Cnockaert M, Swings J (2005) *Aeromonas culicicola* Pidiyar et al. 2002 is a later subjective synonym of *Aeromonas veronii* Hickman-Brenner et al. 1987. Syst Appl Microbiol 28: 604–609. 16156118 10.1016/j.syapm.2005.03.012

[19] Esteve C, Valera L, Gutierrez C, Ventosa A (2003) Taxonomic study of sucrose-positive *Aeromonas jandaei*-like isolates from faeces, water and eels: emendation of *A. jandaei* Carnahan et al. 1992. Int J Syst Evol Microbiol 53: 1411–1419. 13130026 10.1099/ijs.0.02504-0

[20] Demarta A, Huys G, Tonolla M, Swings J, Peduzzi R (2004) Polyphasic taxonomic study of “*Aeromonas eucrenophila*-like” isolates from clinical and environmental sources. Syst Appl Microbiol 27: 343–349. 15214640 10.1078/0723-2020-00276

[21] Martinez-Murcia AJ (1991) An automated RNA extraction procedure and application for 16S rRNA sequencing of *Leuconostoc amelobiosum.* . Microbiologia 7: 106–112. 1760135

[22] Martinez-Murcia AJ (1999) Phylogenetic positions of *Aeromonas encheleia*, *Aeromonas popoffii*, *Aeromonas* DNA hybridization group 11 and *Aeromonas* group 501. Int J Syst Bacteriol 49 Pt 4: 1403–1408. 10.1099/00207713-49-4-1403 10555319

[23] Soler L, Yanez MA, Chacon MR, Aguilera-Arreola MG, Catalan V, et al (2004) Phylogenetic analysis of the genus *Aeromonas* based on two housekeeping genes. Int J Syst Evol Microbiol 54: 1511–1519. 15388703 10.1099/ijs.0.03048-0

[24] Martinez-Murcia AJ, Soler L, Saavedra MJ, Chacon MR, Guarro J, et al (2005) Phenotypic, genotypic, and phylogenetic discrepancies to differentiate *Aeromonas salmonicida* from *Aeromonas bestiarum* . Int Microbiol 8: 259–269. 16562378

[25] Saavedra MJ, Figueras MJ, Martinez-Murcia AJ (2006) Updated phylogeny of the genus *Aeromonas* . Int J Syst Evol Microbiol 56: 2481–2487. 17012583 10.1099/ijs.0.64351-0

[26] Saavedra MJ, Perea V, Fontes MC, Martins C, Martinez-Murcia A (2007) Phylogenetic identification of *Aeromonas* strains isolated from carcasses of pig as new members of the species *Aeromonas allosaccharophila* . Antonie Van Leeuwenhoek 91: 159–167. 17080292 10.1007/s10482-006-9107-5

[27] Fenselau C, Demirev PA (2001) Characterization of intact microorganisms by MALDI mass spectrometry. Mass Spectrom Rev 20: 157–171. 11835304 10.1002/mas.10004

[28] Lay JOJ (2001) MALDI-TOF mass spectrometry of bacteria. Mass Spectrom Rev 20: 172–194. 11835305 10.1002/mas.10003

[29] Welker M, Marsalek B, Sejnohova L, von Dohren H (2006) Detection and identification of oligopeptides in *Microcystis* (cyanobacteria) colonies: toward an understanding of metabolic diversity. Peptides 27: 2090–2103. 16678305 10.1016/j.peptides.2006.03.014

[30] Welker M, Moore ER (2011) Applications of whole-cell matrix-assisted laser-desorption/ionization time-of-flight mass spectrometry in systematic microbiology. Syst Appl Microbiol 34: 2–11. 21288677 10.1016/j.syapm.2010.11.013

[31] Gaia V, Casati S, Tonolla M (2011) Rapid identification of *Legionella* spp. by MALDI-TOF MS based protein mass fingerprinting. Syst Appl Microbiol 34: 40–44. 21247716 10.1016/j.syapm.2010.11.007

[32] Hahn D, Mirza B, Benagli C, Vogel G, Tonolla M (2011) Typing of nitrogen-fixing *Frankia* strains by matrix-assisted laser desorption ionization-time-of-flight (MALDI-TOF) mass spectrometry. Syst Appl Microbiol 34: 63–68. 21242047 10.1016/j.syapm.2010.11.009

[33] Hinse D, Vollmer T, Erhard M, Welker M, Moore ER, et al (2011) Differentiation of species of the *Streptococcus bovis/equinus*-complex by MALDI-TOF Mass Spectrometry in comparison to sodA sequence analyses. Syst Appl Microbiol 34: 52–57. 21247715 10.1016/j.syapm.2010.11.010

[34] Sato H, Teramoto K, Ishii Y, Watanabe K, Benno Y (2011) Ribosomal protein profiling by matrix-assisted laser desorption/ionization time-of-flight mass spectrometry for phylogenety-based subspecies resolution of *Bifidobacterium longum* . Syst Appl Microbiol 34: 76–80. 20828962 10.1016/j.syapm.2010.07.003

[35] Benagli C, Rossi V, Dolina M, Tonolla M, Petrini O (2011) Matrix-assisted laser desorption ionization-time of flight mass spectrometry for the identification of clinically relevant bacteria. PLoS One 6: e16424. 21283545 10.1371/journal.pone.0016424 PMC3026826

[36] Donohue MJ, Smallwood AW, Pfaller S, Rodgers M, Shoemaker JA (2007) Differentiation of *Aeromonas* isolated from drinking water distribution systems. Anal Chem 79: 1939–1946. 17269751 10.1021/ac0611420

[37] Donohue MJ, Smallwood AW, Pfaller S, Rodgers M, Shoemaker JA (2006) The development of a matrix-assisted laser desorption/ionization mass spectrometry-based method for the protein fingerprinting and identification of *Aeromonas* species using whole cells. J Microbiol Methods 65: 380–389. 16176841 10.1016/j.mimet.2005.08.005

[38] Martinez-Murcia AJ, Saavedra MJ, Mota VR, Maier T, Stackebrandt E, et al (2008) *Aeromonas aquariorum* sp. nov., isolated from aquaria of ornamental fish. Int J Syst Evol Microbiol 58: 1169–1175. 18450708 10.1099/ijs.0.65352-0

[39] Demarta A, Tonolla M, Caminada A, Beretta M, Peduzzi R (2000) Epidemiological relationships between *Aeromonas* strains isolated from symptomatic children and household environments as determined by ribotyping. Eur J Epidemiol 16: 447–453. 10997832 10.1023/a:1007675424848

[40] Yamamoto SHS (1995) PCR amplification and direct sequencing of *gyrB* genes with universal primers and their application to the detection and taxonomic analysis of *Pseudomonas putida* strains. Appl Environ Microbiol 61: 1104–1109. 7793912 10.1128/aem.61.3.1104-1109.1995 PMC167365

[41] Kupfer M, Kuhnert P, Korczak BM, Peduzzi R, Demarta A (2006) Genetic relationships of *Aeromonas* strains inferred from 16S rRNA, *gyrB* and *rpoB* gene sequences. Int J Syst Evol Microbiol 56: 2743–2751. 17158971 10.1099/ijs.0.63650-0

[42] Kumar S, Jakobsen IB, Nei M, Bioinformatics (2001) MEGA2:cmolecular evolutionary genetics analysis software. 17 1244–1245. 10.1093/bioinformatics/17.12.1244 11751241

[43] Martinez-Murcia A, Monera A, Alperi A, Figueras MJ, Saavedra MJ (2009) Phylogenetic evidence suggests that strains of *Aeromonas hydrophila* subsp. *dhakensis* belong to the species *Aeromonas aquariorum* sp. nov. Curr Microbiol 58: 76–80. 18839248 10.1007/s00284-008-9278-6

