# Supplementary material for: A Rapid MALDI-TOF MS Identification Database at Genospecies Level for Clinical and Environmental Aeromonas Strains
Source: PLoS One. 2012 Oct 31;7(10):e48441. doi: 10.1371/journal.pone.0048441 (PMC3485216; doi:10.1371/journal.pone.0048441)
Supplement: Table S1 — Strains used in this study. (DOC) [file pone.0048441.s001.doc]

| **Species** | **HG** | **Strains** | | | |
| --- | --- | --- | --- | --- | --- |
|  |  | **Designation** | **Our designation** | **Other designation** | **GenBank accession number** |
| *A. hydrophila* | 1 | ATCC 7966T | 128 | CECT839T=DSM30187T=Popoff 543T | AF417622 |
|  |  |  | F458 |  | AJ868393 |
|  |  |  | F589 |  | =F458 |
|  |  |  | F567A |  | =F458 |
| *A. hydrophila* subsp*. dhakensis* |  | CIP 107500T | 225 |  | AM262163 |
| *A. hydrophila* subsp*. ranae* |  | CIP 107985T | 226 |  | AM262162 |
| *A. bestiarum* | 2 | ATCC 51108T | 157 | CECT4227T=CDC953376T=LMG3751T | AF417623 |
|  |  |  | F530D |  | AJ868370 |
|  |  |  | F666I |  | AJ868371 |
| *A. salmonicida* biotype *salmonicida* | 3 | ATCC 33658T | 150 | CECT839T=LMG3780 | AY294485 |
|  |  |  | V155 |  | AJ868365 |
|  |  |  | V1 |  | AJ868363 |
|  |  |  | V32 |  | AJ868364 |
|  |  | JF2267 | 222 |  | JX032697 |
|  |  | JF2267 | 253=222 |  | JX032697 |
|  |  | JF3327 | 255 |  | JX032698 |
|  |  | JF3507 | 256=150 | ATCC33658T=NCIMB1102T | AY294485 |
|  |  | JF4117 | 261 |  | JX032699 |
| *A. salmonicida* *smithia* |  | JF3117 | 258 | NCIMB13210R=ATCC49393T | AM262159 |
|  |  | JF4097 | 260 |  | FN394064 |
|  |  | JF4439 | 262 |  | =260 |
| *A. salmonicida* *achromogenes* |  | NCIMB 1110T | 250 |  | AM262161 |
|  |  | JF2997 | 254 |  | JX032700 |
|  |  | JF3116 | 257=250 | NCIMB1110T | AM262161 |
| *A. salmonicida masoucida* |  | JF3118 | 259 | ATCC27013T | AM262160 |
| *A. salmonicida* *pectinolytica* |  | DSM 12609T | 251 |  | AM262158 |
|  |  |  | V183 |  | AJ868369 |
|  |  |  | V23 |  | AJ868368 |
|  |  |  | F553E |  | AJ868367 |
|  |  |  | V130 |  | AJ868366 |
| *A. caviae* | 4 | ATCC 15468T | 129 | CECT838T=LMG3775 | AJ868400 |
|  |  |  | V30 |  | AJ868397 |
|  |  |  | V83 |  | AJ868399 |
|  |  |  | V97 |  | AJ868395 |
|  |  |  | V39 |  | AJ868398 |
|  |  |  | F507C |  | AJ868396 |
| *A. hydrophila* subsp. *anaerogenes* **CAVIAE** |  | CIP76.15T | 227 |  | AM262164 |
| *A. media* | 5A | CDC 0862-83 | 151 |  | AJ868380 |
|  |  |  | V168 |  | AJ868381 |
|  |  |  | V69 |  | AJ868382 |
|  |  |  | V47 |  | AJ868379 |
|  | 5B | ATCC 33907T | 125 |  | AF417627 |
|  |  |  | V6 |  | AJ868384 |
|  |  |  | V15 |  | =V6 |
|  |  |  | F674P |  | AJ868383 |
| *A. eucrenophila* | 6 | ATCC 23309T | 123 | CECT4224T=LMG3774T=NCIMB74T | AF417629 |
|  |  | LMG17059 | 185 |  | AM116970 |
|  |  | LMG16179 | 184 |  | AM179829 |
|  |  | LMG 13058 | 181 |  | JX032696 |
|  |  | LMG13057 | 180 |  | AY101820 |
|  |  | LMG13687 | 183 |  | AY101813 |
|  |  | LMG 13060 | 182 |  | JX032695 |
|  |  |  | F729F |  | AJ868377 |
| *A. encheleia* | 16 | CECT 4342T | 179 | ATCC51929T=NCIMB13442T | AF417635 |
|  |  | LMG16331 | 190 |  | AM179830 |
|  |  | LMG13062 | 187 |  | AY101809 |
|  |  | ATCC 51930 | 194 | CECT4341=LMG16329 | AY101804 |
|  |  | LMG13061 | 186 |  | DQ411489 |
|  |  | LMG13691 | 188 |  | DQ411486 |
| *A. sobria* | 7 | ATCC 43979T | 130 |  | AY101781 |
| *A. jandaei* | 9 | ATCC 49568T | 153 | CECT4228=LMG12221=CDC0787-80 | AJ868391 |
| *A. veronii* biotype *sorbia* | 8 | ATCC 51106 | 152 | CDC0437-84 | AF417632 |
| *A. veronii* biotype *veronii* | 10 | JF2853 | 220 |  | AJ868389 |
|  |  | JF2689 | 216 |  | AJ868389 |
|  |  | JF2638 | 215 |  | AJ868387 |
|  |  | ATCC 35624T | 127 |  | AF417626 |
| *Aeromonas* sp. | 11 | ATCC 35941T | 154 | CETC4253T=NCIMB13014T | AJ964951 |
|  |  |  | F544A |  | AJ868374 |
| *A. schubertii* | 12 | ATCC 43700T | 126 | CETC4240T=CDC2446-81T=LMG9074 | AJ868402 |
| *Aeromonas* sp. Group | 13 | ATCC 43946T | 221 | CETC4254T=LMG17321T=CDC244681T | AF417630 |
| *A. trota* | 14 | ATCC 49657T | 155 | CETC4255=LMG12223 | AY851131 |
| *A. allosaccharophila* | 15 | ATCC 51208T | 178 | CECT4199T=LMG14059T | AY101777 |
| *A. popoffi* | 17 | LMG 17541T | 167 | CECT5176T | AJ868372 |
|  |  | LMG 17542 | 168 |  | JX032694 |
|  |  | LMG 17544 | 170 |  | AY101821 |
|  |  |  | F600C |  | AJ868373 |
|  |  |  | F548B |  | =533E |
|  |  |  | F533E |  | AJ868367 |
| *A. culicicola* |  | CIP 107763T | 212 |  | AJ868386 |
|  |  |  | F474 |  | AJ868388 |
| *A. molluscorum* |  | LMG 22214T | 246 | CECT5864=DSM17090 | AY987538 |
| *A. simiae* |  | CIP 107798T | 214 | IBSS6874T | DQ411480 |
| *A. sharmana* |  | DSM 17445T | 247 |  | AM490259 |
| *A. tecta* |  |  | Aer 58 |  | AJ964955 |
|  |  |  | Aer 106 |  | AJ964954 |
|  |  |  | F713G |  | AJ964953 |
|  |  |  | F518 |  | AJ964952. |
|  |  |  | F713E |  | AJ868376 |
| *A. bivalvium* |  | 665N | 248 |  | DQ504430 |
|  |  | 868E(T) | 249 |  | EF465525 |
| *A. aquariorum* |  | DSM 18362 | 252 |  | HQ442712 |

ATCC, American Type Culture Collection, Manassas, VA, USA; AMC, Amy M. Carnahan, University of Maryland, College Park, MD, USA

CDC, Centers for Disease Control and Prevention, United States Public Health Service, Atlanta, GA, USA; CECT, Coleccion Espanola de Cultivos

Tipo, Universidad De Valencia, Valencia, Spain; LMG, Culture collection of the Laboratorium voor Microbiologie Gent, Microbiologie Gent, Gent

Belgium; NMRC, Naval Medical Research Center, Silver Springs, MD, USA; SMHC, Southern Maryland Hospital Center, Clinton, MD, USA

WR, Walter Reed Army Medical Center, Washington DC, USA.

a Type strain
